# Knowledge status and sampling strategies to maximize cost-benefit ratio of studies in landscape genomics of wild plants

**DOI:** 10.1038/s41598-020-60788-8

**Published:** 2020-02-28

**Authors:** Alesandro Souza Santos, Fernanda Amato Gaiotto

**Affiliations:** Applied Ecology & Conservation Lab, Santa Cruz State University - Rodovia Ilhéus-Itabuna, km 16, Ilheus, BA ZIP Code: 45662-901 Brazil

**Keywords:** Ecological genetics, Plant genetics, Molecular ecology

## Abstract

To avoid local extinction due to the changes in their natural ecosystems, introduced by anthropogenic activities, species undergo local adaptation. Landscape genomics approach, through genome–environment association studies, has helped evaluate the local adaptation in natural populations. Landscape genomics, is still a developing discipline, requiring refinement of guidelines in sampling design, especially for studies conducted in the backdrop of stark socioeconomic realities of the rainforest ecologies, which are global biodiversity hotspots. In this study we aimed to devise strategies to improve the cost-benefit ratio of landscape genomics studies by surveying sampling designs and genome sequencing strategies used in existing studies. We conducted meta-analyses to evaluate the importance of sampling designs, in terms of (i) number of populations sampled, (ii) number of individuals sampled per population, (iii) total number of individuals sampled, and (iv) number of SNPs used in different studies, in discerning the molecular mechanisms underlying local adaptation of wild plant species. Using the linear mixed effects model, we demonstrated that the total number of individuals sampled and the number of SNPs used, significantly influenced the detection of loci underlying the local adaptation. Thus, based on our findings, in order to optimize the cost-benefit ratio of landscape genomics studies, we suggest focusing on increasing the total number of individuals sampled and using a targeted (e.g. sequencing capture) Pool-Seq approach and/or a random (e.g. RAD-Seq) Pool-Seq approach to detect SNPs and identify SNPs under selection for a given environmental cline. We also found that the existing molecular evidences are inadequate in predicting the local adaptations to climate change in tropical forest ecosystems.

## Introduction

Anthropogenic activities are transforming natural systems, drastically changing the environmental conditions in a way which poses major threat to global biodiversity^1,2^. Anthropogenic modifications can lead to reduction and fragmentation of natural environments, or to changes in climatic conditions^3–5^. Species respond to such changes in the natural habitat, by (i) phenotypic plasticity, (ii) migrating from their natural habitats, in search of conditions, fit for survival and having ample resources, or (iii) adapting to the new environment to avoid local extinction (local adaptation)^4,6–8^.

A species is said to exhibit local adaptation, when individuals on an average have a superior fitness in their home environment, compared to a transplanted individual^8–11^. Therefore, local adaptation is driven by the action of natural selection, which acts on the individual’s phenotypes, determining the characteristics that will be favored under certain environmental conditions^9,12,13^. Historically, local adaptation has been studied either through translocation experiments (between environments) or trials in the greenhouse under controlled environmental conditions^8,14,15^. The drawbacks of these two approaches include the requirement of ample financial resources and time, which is generally scarce for studies involving long-lived species such as trees^14,15^. Landscape genomics, a study that identifies genetic variations that confer local adaptation, is used to remedy these limitations^16^. In this approach, significant differences in allele frequency between populations of the target species, indicates that individuals in the population are experiencing selection pressure; possibly in response to change in some environmental factor, such as changes in soil type, radiation, water stress, and temperature^17–20^. Landscape genomics studies analyze frequency distribution changes in molecular markers, such as single nucleotide polymorphism (SNPs)^14,21^ in relation to given environmental factors. The SNPs are commonly used in wild local adaptation studies because their location and functional annotations are known and they are widely distributed throughout the genome^22,23^. Additionally, the advent of high-throughput sequencing technology (HTS) has made the sequencing of millions of SNPs from the genome, possible, at moderate cost and it is not time intensive^24,25^. Thus, through the use of landscape genomics, it is now possible to find correlations between genomic regions and the variable environmental characteristics^15,21^. Therefore, this approach is used to pinpoint the environmental change in nature, that affects ecology (e.g.: climatic changes) of a species and can influence its adaptive genetic potential^26–28^. Thus, landscape genomics approach is useful in predicting the responses of species to environmental heterogeneity and variable landscape factors^29^.

In landscape genomics studies, outlier loci method has been used in tandem with genome–environment association (GEA) method, to evaluate local adaptation in natural populations^21^. In the outlier loci method, changes in among-population frequency distribution of given allele(s)/loci, significantly different from that seen in the absence of natural selection, are identified, and these alleles/loci are considered to be under natural selection pressure^15,21^. While, in the GEA analysis, occurrence of high correlation between the allele frequencies with one or more environmental variables is considered an indicator of local adaptation^10,30^. However, as the outlier method does not specify the environmental forces at work on the locus under selection, studies using landscape genomics approach use the GEA results^31,32^ to fix the causative environmental factor. For this reason, in this study we tried to systematize and discuss the results obtained from GEA in wild populations, sampled *in situ*.

Considering that landscape genomics is a relatively new discipline, it requires refinement within the scope of sampling design, for GEA studies^33–37^. Studies, based on simulations, have predicted that the number of SNP markers used and the number of populations sampled influence the inferences from landscape genomics analysis^38,39^. However, a recent review article, evaluated the impacts of sampling design on inferences from empirical studies on landscape genomics and it does not take into account the particularities of tropical regions^37^. In a way, our approach complements that of Ahrens *et al*., as they focused on the limitations of the different techniques, the lack of standardization and non-availability of information in conducting these studies. However, unlike others, our study, by reporting on empirically observed patterns in landscape genomics, aims to shape sampling design strategies for improving the cost-benefit ratio of future works, conducted in laboratories using population genomics for the first time. Guidelines put forth in this study will be helpful, in particular, in subsidizing the costs of studies being carried out in the stark socioeconomic realities of tropical regions, which are global biodiversity hotspots under grave anthropogenic threat.

## Methodology

**Figure 1:**
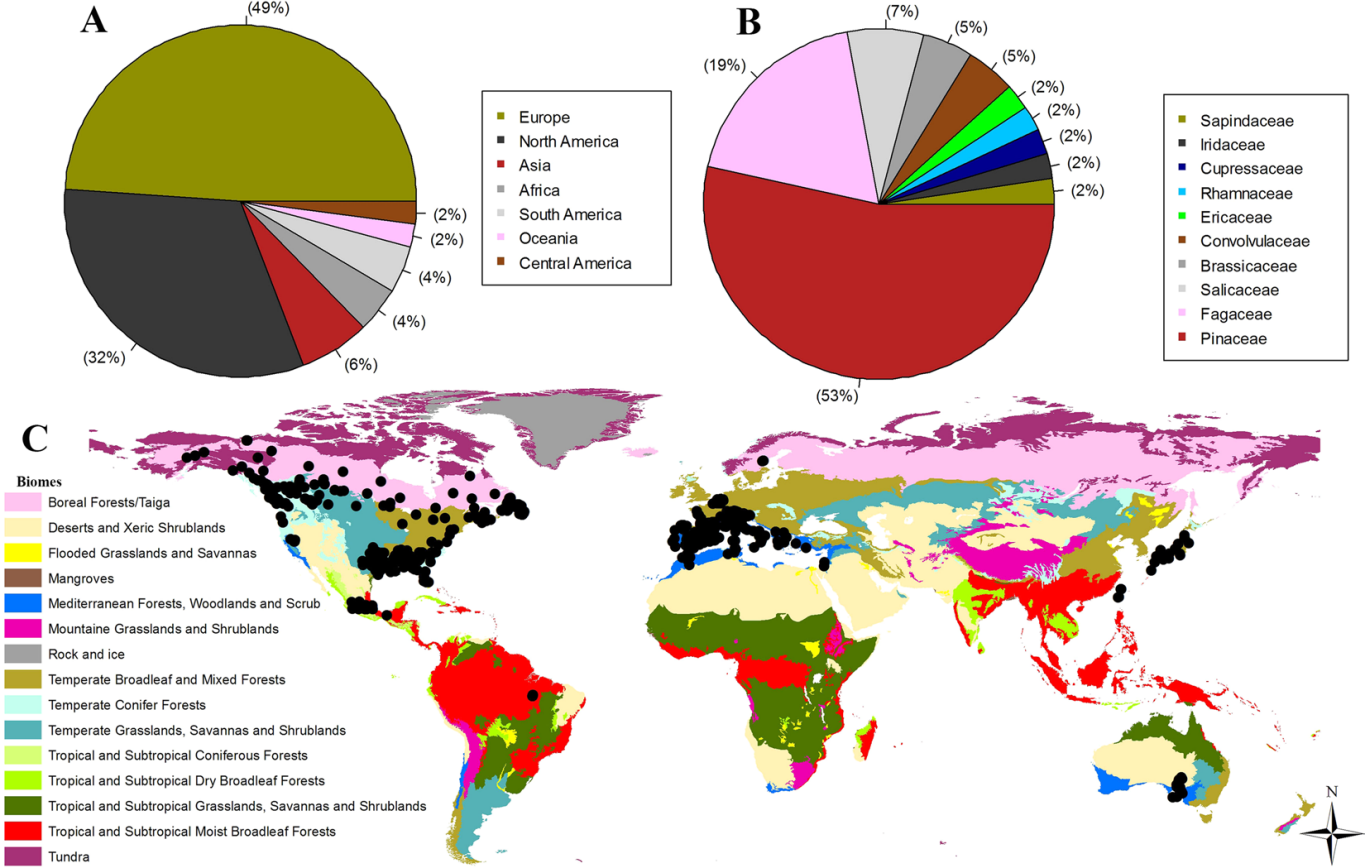
Geographical, ecological and species distribution found in the 35 research articles, included in this study, which evaluated local adaptation in wild plant populations in their natural occurrence area using SNPs markers, are shown; (**A**) Percentage of studies published per continent; (**B**) Percentage of studies distributed per plant family; (**C**) The points distributed on the map indicating the sampling of the studies in the different terrestrial biomes.

To perform this work, all research articles, published to date (at the time of writing, September 2018), which evaluated local adaptations in populations of wild plants were pooled. The papers were analyzed for (i) number of populations sampled, (ii) number of individuals sampled per population, (iii) total number of individuals sampled, and (iv) number of SNPs used. Care was taken to ensure that all data were obtained in empirical studies assessing local adaptation in wild plant populations. The Scopus (https://www.scopus.com) and Google Scholar (http://scholar.google.com.br/) databases were searched for title, abstract, and articles using the following keywords: environmental variables and SNPs, landscape genetics and SNPs, spatial analysis and SNPs, landscape genomics and SNPs, population genomics and SNPs, adaptive genetic variation, and local adaptation and SNPs. Search results were filtered to remove papers and reviews from clinical, biomedical, veterinary, and immunology areas. Then, a second filter was applied to eliminate the articles containing the following words: animal, fish, ecology of freshwater, marine biology, entomology, and zoology. Finally, papers using simulations, or involving exotic and crop species in plantations, or using greenhouse experiments were removed and the remaining 35 empirical studies on *in situ* wild plants were selected for the present work. Using this data set and ArcGIS (10.2), a map was created depicting the distribution of the localities where researches were performed, in the different terrestrial biomes (Fig. 1). A double check was done at this stage to ensure if all the 35 papers chosen for this study, had evaluated *in situ* local adaptation in wild plant populations, using SNPs markers. The following information from the papers: (1) authors names; (2) publication year, (3) country of study and geographical coordinates; (4) botanical family studied; (5) species; (6) total number of individuals; (7) number of populations; (8) number of SNPs used; (9) number of SNPs under selection; and (10) method used to generate the SNPs data, was compiled.

For this study, we considered as SNPs under selection, the SNPs from the pooled data that had a significant association with a geoclimatic variable, such as temperature, precipitation, latitude, longitude, elevation, evapotranspiration, and drought. They were used as a proxy to detect the potential for natural selection in wild plant populations. The number of SNPs under selection could be influenced by the methods employed to analyze the correlation between allelic frequency and environmental variables^38^, therefore most articles in the literature make inferences from such studies, using conservative criteria^34,35,40^. It is known that each method has different types of limitations and caveats (e.g., vary in the rates of false positives or of false negatives), which can cause errors in the estimates and consequently in the estimate of the number of SNPs under selection^38^. For this reason, we chose to be conservative, using only the results obtained either simultaneously by at least two methods of analysis or strictly controlled for rates of false positives or false negatives (e.g., studies that controlled the population structure in the analyses). Thus, although limitations of the different methodologies used were not fully circumvented, we believe that the results reported in our mini-review, portrays the findings of the literature, in a way seeking to minimize, as much as possible, the biases inherent to the different analysis.

The methods used to generate the SNPs data were subdivided into Random, Random Pool-Seq, Targeted, and Targeted-Pool-Seq categories. The Random category included studies that used random regions of DNA and individualized sequencing for the preparation of libraries. The Random-Pool-Seq category is distinct from Random, in that the data is sequenced with the Pool-Seq technique (Pool-Seq, an equimolar amount of DNA is taken from each individual from a population and pooled for sequencing). In the Targeted category, we included studies that used only specific gene regions or expressed sequence tags with individualized sequencing. The Targeted Pool-Seq category differed from Targeted, such that the data is sequenced by Pool-Seq technique mentioned above.

Influence of sample size on number of SNPs under selection was inferred by determining the average of the individual number of samples from each population which was arrived at by dividing the total number of individuals and the number of populations in each study. Additionally, the percentage of SNPs under selection was calculated to estimate the number of genes under natural selection in the genome. Then, a descriptive statistical analysis (minimum, maximum, mean, and standard) was performed with all the variables in R program (http://www.r-project.org/).

To evaluate the influence of each variable on the number of SNPs under selection, the linear mixed effects model of the lme4 package of R program^41^ was used. The variables in this study, such as the number of populations, number of individuals, average number of individuals sampled per population, and number of SNPs used were investigated to determine their influence on the number of SNPs under selection and, consequently, the detection of natural selection in plant species. As this type of model requires the fulfillment of the assumptions of normality and homoscedasticity of the residues, we performed the scaling of each variable separately. We used the rank function in R program to reduce the amplitude of our data, meet the assumptions, and obtain the goodness of fit of the statistical models. For each variable, an independent model was created by considering each variable as the fixed effect and the others as the random effect, such that:

$$\begin{array}{c}{{\rm{n}}}^{{\rm{o}}}\,{\rm{of}}\,{\rm{SNPs}}\,{\rm{under}}\,{\rm{selection}} \sim {{\rm{n}}}^{{\rm{o}}}\,{\rm{of}}\,{\rm{SNPs}}\,{\rm{used}}+(1|{{\rm{n}}}^{{\rm{o}}}\,{\rm{of}}\,{\rm{individuals}})\\ +(1|{{\rm{n}}}^{{\rm{o}}}\,{\rm{of}}\,{\rm{pop}})+(1|{{\rm{n}}}^{{\rm{o}}}\,{\rm{of}}\,{{\rm{ind}}}_{-}\,{\rm{pop}}).\end{array}$$

where, the number of SNPs under selection is the response variable; number of SNPs used is the fixed-effect variable; the terms in parentheses are the random effect variables and the number 1 indicates that the intercept is random between the observations of each variable.

The linear mixed effects model in the lme4 package does not provide the results as the coefficient of determination (r²), due to theoretical problems or difficulty of implementation. For this reason, we used the marginal r² to calculate the amount of variance explained by the fixed-effect variables in each model^42^. Finally, we used the function lm in R to establish the simple linear regression analysis of the number of SNPs used in the function of the total number of individuals. To make the graphs with the significant results of all the analyses, we used the ggplot2 package in R^43^.

## Results

Based on the 35 selected empirical articles, we analyzed 44 observations involving 36 different species for the number of SNPs under selection (Table 1). These studies were conducted mainly in Europe and North America (Fig. 1A), in ten plant families, dominance by Pinaceae (53% of the species) (Fig. 1B), distributed mainly in biomes of temperate broadleaf and mixed forest, temperate grasslands and Mediterranean forests (Fig. 1C). The total number of individuals ranged from 22 to 2,574, and the number of SNPs used varied between 33 and 2,091,957. However, the number of SNPs under selection (SNPs with significant association with some geoclimatic variable) varied from 2 to 2,522 with the percentage of SNPs under selection ranging from 0.02 to 78.14 (Table 1).

**Table 1:** Biological models and descriptive statistics calculated in 35 studies that assessed local adaptation in wild plant populations *in situ* using markers SNPs.

Author	Species	Pop	Ind	Ind_Pop	SNPs	SNPs_GEA	SNPs_GEA*	Method
Eckert *et al*.^57^	*Pinus taeda*	54	682	12.63	1730	118	6.82	Targeted
Mosca *et al*.^58^	*Abies alba Mill*	37	1183	31.97	249	3	1.20	Targeted
Mosca *et al*.^58^	*Larix decidua Mill*	24	920	38.33	267	5	1.87	Targeted
Keller *et al*.^59^	*Populus balsamifera L*	31	443	14.29	335	11	3.28	Targeted
Prunier *et al*.^60^	*Picea mariana*	41	593	14.46	47	9	19.15	Targeted
Mosca *et al*.^58^	*Pinus cembra L*	24	860	35.83	459	11	2.40	Targeted
Mosca *et al*.^58^	*Pinus mugo*	27	935	34.63	693	14	2.02	Targeted
Fischer *et al*.^23^	*Arabidopsis halleri*	5	100	20.00	2091957	1037	0.05	Random-Pool-Seq
Bashalkhanov *et al*.^31^	*Picea rubens*	5	900	180.00	33	6	18.18	Targeted
Mosca *et al*.^61^	*Abies alba Mill*	36	1108	30.80	231	5	2.16	Targeted
Tsumura *et al*.^62^	*Cryptomeria japonica*	14	186	13.29	3930	25	0.64	Targeted
Mosca *et al*.^61^	*Larix decidua Mill*	22	824	37.50	233	7	3.00	Targeted
Modesto *et al*.^63^	*Quercus suber L*	16	96	6.00	44	5	11.36	Targeted
Scalfi *et al*.^64^	*Picea abies [L.] Karst)*	12	300	25.00	227	2	0.88	Targeted
Cullingham *et al*.^46^	*Pinus contorta var. latifolia*	13	368	28.31	399	22	5.51	Targeted
Cullingham *et al*.^46^	*Pinus banksiana*	4	100	25.00	399	8	2.01	Targeted
Geraldes *et al*.^65^	*Populus trichocarpa*	25	424	16.96	28135	58	0.21	Targeted
H De Kort *et al*.^66^	*Frangula alnus subsp. alnus*	25	619	24.76	183	143	78.14	Targeted
Hamlin *et al*.^67^	*Iris hexagona*	8	92	11.50	750	70	9.33	Random
Eckert *et al*.^68^	*Pinus lambertiana*	10	241	24.10	475	14	2.95	Targeted
Jaramillo-Correa *et al*.^69^	*Pinus pinaster Aiton*	36	772	21.44	266	18	6.77	Targeted
Roschanski *et al*.^13^	*Abies alba Mill*	4	376	94.00	267	8	3.00	Targeted
Christmas *et al*.^25^	*Dodonaea viscosa ssp. Angustissima*	17	89	5.24	8462	93	1.10	Targeted
Pluess *et al*.^10^	*Fagus sylvatica*	79	234	2.96	144	16	11.11	Targeted
Gugger *et al*.^30^	*Quercus lobata*	12	22	1.83	220427	79	0.04	Targeted
Sork *et al*.^20^	*Quercus lobata*	13	45	3.46	195	8	4.10	Targeted
Rellstab *et al*.^12^	*Quercus robur*	24	465	19.38	3576	181	5.06	Targeted-Pool-Seq
Rellstab *et al*.^12^	*Quercus petraea*	18	350	19.44	3576	224	6.26	Targeted-Pool-Seq
Rellstab *et al*.^12^	*Quercus pubescens*	17	326	19.18	3576	304	8.50	Targeted-Pool-Seq
Di Pierro *et al*.^18^	*Norway spruce*	24	826	34.40	214	10	4.67	Targeted
Di Pierro *et al*.^18^	*Picea abies [L.] Karst*	23	826	35.91	214	7	3.27	Targeted
Mosca *et al*.^19^	*Pinus cembra L*	18	678	37.67	455	74	16.26	Targeted
Mosca *et al*.^19^	*Pinus mugo*	20	673	33.65	663	60	9.05	Targeted
Rajora *et al*.^28^	*Pinus strobus*	29	638	22.00	44	2	4.55	Targeted
Di Pierro *et al*.^70^	*Picea abies [L.] Karst*	18	687	38.17	175	19	10.86	Targeted
Lind *et al*.^71^	*Pinus albicaulis Engelm*	8	244	30.50	116231	1780	1.53	Random
Fahrenkrog *et al*.^72^	*Populus deltoides*	50	168	3.36	79969	2.522	3.15	Targeted-Pool-Seq
Frachon *et al*.^54^	*Arabidopsis thaliana*	168	2.574	15.32	1638649	300	0.02	Random-Pool-Seq
Lanes *et al*.^73^	*Ipomoea cavalcantei*	1	122	122.00	34102	2239	6.57	Random
Lanes *et al*.^73^	*Ipomoea maurandioides*	4	254	63.50	23181	1814	7.83	Random
Shih *et al*.^56^	*Keteleeria davidiana var. formosana*	5	62	12.40	13914	15	0.11	Random-Pool-Seq
Martins *et al*.^74^	*Quercus rugosa Née*	17	103	6.06	5354	97	1.81	Random-Pool-Seq
Ruiz Daniels *et al*.^75^	*Pinus halepensis Mill*	46	1.326	28.83	294	7	2.38	Targeted
Alam *et al*.^76^	*Vaccinium vitis-idaea subsp. Minus*	56	56	1.00	1586	132	8.32	Random
**Descriptive statistics**	**Minimum**	**1**	**22**	**1.00**	**33**	**2**	**0.02**	
	**Maximum**	**168**	**2574**	**180**	**2091957**	**2522**	**78.14**	
	**Mean**	**25.91**	**520.2**	**29.48**	**97420**	**263.2**	**7**	
	**Standard deviation**	**27.28**	**471.82**	**32.31**	**395181.4**	**614**	**11.99**	

(Ind, Number of individuals; Ind_Pop, Average number of individuals per population; SNPs, Number of used SNPs; Pop, Number of populations; SNPs_GEA, Number of SNPs under selection; SNPs_GEA*, Percentage of SNPs under selection; Method, Method used to generate the SNP data).

**Figure 2:**
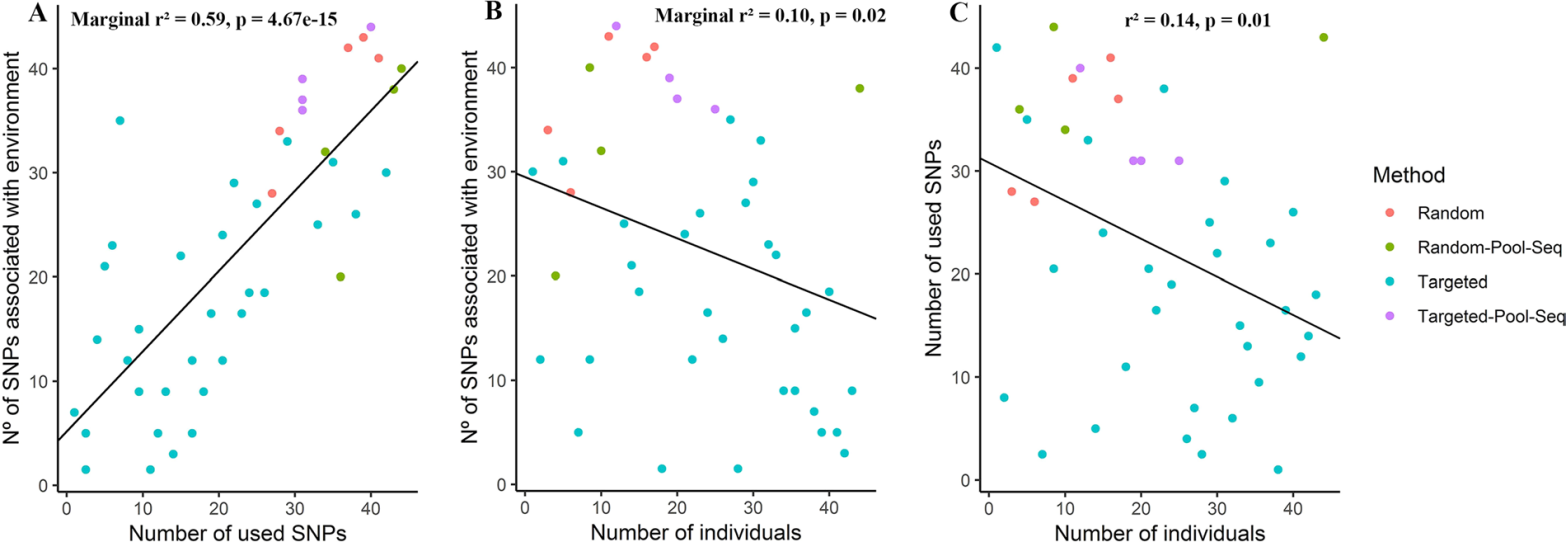
Relation between sampling design and the detection of SNPs with environmental association **(A)** number of SNPs with environmental association as a function of the number of SNPs used in the studies; **(B)** number of SNPs with environmental association as a function of the total number of individuals in each study; **(C)** number of SNPs used as a function of the total number of individuals in each study. The colors of the dots represent the different methods used in the studies (Random used random regions of DNA and individualized sequencing; Random Pool-Seq used random regions of DNA and Pool-Seq technique; Targeted used only gene regions or expressed sequence tags and individualized sequencing; Targeted Pool-Seq used only gene regions or expressed sequence tags and sequencing in Pool-Seq).

In the analysis using the linear mixed-effects models, we verified the effect of each variable (related to sample size and SNP number) individually on the number of SNPs under selection, whereas the others were used as random effect, as mentioned in the methodology section. On entering, the number of populations, as fixed effect variable, the model showed no significant relation with the number of SNPs under selection (marginal r² = 0.011, p = 0.38). A similar result was obtained when the mean number of individuals was used as the fixed effect variable (Marginal r² = 0.041, p = 0.16). However, when the number of SNPs was used as the fixed effect variable, a positive and significant relationship was observed with the number of SNP under selection (Fig. 2A). When the total number of individuals was used as a fixed effect variable in the model, a negative and significant relation was observed in relation to the number of SNPs under selection (Fig. 2B). Subsequently, it was verified that the total number of individuals had a negative and significant relation with the number of SNPs used (Fig. 2C).

## Discussion

The data obtained in the systematic review of studies in wild plant populations showed great heterogeneity in the number of individuals, populations, and quantity of SNPs. Our meta-analyses revealed that the total number of individuals and number of SNPs used are of fundamental importance in detecting signs of natural selection in wild plant populations. We also found an enormous knowledge gap in neotropics, underscoring the fact that predicting the response of the tropical forests to geoclimatic changes based on available data is not possible. Most of the studies had been conducted in the biomes of temperate and mixed forests of the European and North American habitats. However, we had to use them to apply the results in the recommendation for other environments, in order to help in decentralizing studies that are performed almost exclusively in the North hemisphere. Thus, it becomes evident the need to increase the possibilities of develop general methodologies guidelines that support decision making about sampling based on what we have available in the literature. Therefore, although our results were mostly based on species from temperate environment, we believe that they can be generalized to global conclusions. For this reason, our discussion focused on tropical regions, as they are recognized as a biodiversity hotspot and having really few studies with landscape genomics.

We found few empirical studies evaluating SNPs under natural selection, with only 36 plants species under analysis. From those studies, we registered ten families, where 53% of the species belonged to the Pinaceae family. This result evidences the poor understanding of local adaptation processes for different species. Considering that tropical forests have approximately 11,371 species of trees^44^, we have a limited vision to predict how species may respond to anthropogenic changes like climate change, fragmentation, and forest loss^45,46^. In addition, ~81% of studies were conducted in the European continent or in North America. Although much of the plant diversity is located in tropical regions^44,47^, the ability of these species to adapt *in situ* in response to environmental changes has not been studied^48^. We believe that this lack of knowledge for the tropics is a reflection of the socioeconomic conditions of this region as well as the lack of specialists in the area since population genomics is relatively recent and the first studies in the tropics are just emerging (see Table 1). Considering that tropical regions could be severely affected by climate change^4^, our results have shown the great need for studies that seek to understand how geoclimatic variables influence local adaptation. Through these studies, it will be possible to predict how tropical species will respond to climate change and assist in their conservation and management^29,32,35,45^.

With our systematic review of empirical studies, we have evidenced that the number of populations and mean number of individuals in a population do not influence the ability to detect natural selection. This emphasizes the importance of sampling, at the best, the whole extent of the area that is being influenced by the environmental variable of interest, rather than focusing on the number of populations sampled or on the mean number of individuals per population. If many populations, from similar environments, are sampled, the probability of detecting local adaptation does not increase^39^. On the other hand, even if few populations are sampled in contrasting environmental conditions, it would increase the power to detect local adaptation^39^. Thus, it will be more effective to cover the environmental heterogeneity, avoiding efforts both in the expansion of the number of populations and in the average number of individuals per population.

The linear mixed-effect model showed that the number of SNPs used explained 59% of the variation in the number of SNPs under selection and therefore can positively influence the detection of natural selection signals. This could occur because mostly a low percentage of genes in a genome are under natural selection (Table 1); therefore, increasing the genome sampling, also increases the chance of identifying SNPs under selection. Interestingly, a general pattern observed was that studies using Random, Targeted Pool-Seq, and Random Pool-Seq methodologies used more SNPs and had a greater number of SNPs under selection. Therefore, it would be beneficial if future works used one of these methodologies, especially Random Pool-Seq and Targeted Pool-Seq, to optimize the cost-benefit ratio. In this context, studies that aim to evaluate the indicators of natural selection in populations of wild plants could use the Targeted Pool-Seq approach, in cases where genomic information of the species is available (e.g., genome size and gene annotation). By using targeted sequencing in DNA pools, cost benefit ratio of such studies would be increased. In contrast, for the species with little or no genomic information available and for laboratories that are working on landscape genomics for the first time, Random Pool-Seq is the best strategy to increase the chances of detecting SNPs under selection. An interesting alternative to make landscape genomics financially feasible would be the use of grouped sequencing of individuals by techniques like RADseq that do not require any prior knowledge of the genome^49^. While it is still advantageous to use the Pool-Seq technique which can increase accuracy in allele frequency estimates, compared to individualized sequencing, making it an excellent tool to be used in landscape genomic studies^50–52^. Moreover, it also allows to reconcile the sampling of a large number of individuals needed in population genomics, with thousands or millions of SNPs, to evaluate adaptation in natural populations^12,23,53,54^. It is important to note that the Pool-Seq technique has some limitations, for example, not providing information for each individual separately^52^. Therefore, the use of this technique is only suitable for studies aiming for population inferences without the need of information about individuals.

By fixing the variable in the linear mixed effects model as total number of individuals, we found that the total sample size was inversely related to the number of SNPs under selection and to the number of SNPs used. Although significant, these correlations explain only 10% of the variation in the number of SNPs under selection and 14% in the number of SNPs used. However, much of the variation in the number of SNPs under selection (59%) could be explained by fixing the variable as the number of SNPs used. We therefore, believe that the significantly negative correlation between the total number of individuals and the number of SNPs under selection is a reflection of the inverse relationship observed between the total number of individuals and the number of SNPs used. Thus, the negative relation seen between the total number of individuals and the number of SNPs under selection is an artifact generated by the lower capacity detection of natural selection when the number of SNPs used decreases. The negative relationship observed between the number of individuals sampled and the number of SNPs used could be due to incomplete genomic information because of the costs associated with sequencing of whole genomes^53^. Among species for which some genomic information is available, size consideration of the genome becomes important; because the larger genomes may need more number of SNPs to be sampled to give a full perspective, thus impacting the number of individuals that can be analyzed given the budgetary constraints. Also, investing a huge portion of the budget for sampling in the field to increase the sample size, would affect the laboratory stages of the study by limiting resource allocation. Thus, the negative relation found in this study, can be related to the costs of individual sequencing of each sample. In population genomics studies, the conflicting demands between the total number of individuals and the number of SNPs used is circumvented by using the Pool-Seq technique^53^ (Table 1). This technique has also been used in plant species with incomplete or total absence of genomic information^52^. This indicates that the same strategy can be used in the landscape genomics studies of species in tropical regions, which are major biodiversity hotspots of the world^44,47^ with little or no genomic information available.

## Guidance for Study of Local Adaptation in Wild Plant Species

Our study involving systematic review of empirical studies and meta-analysis of data and results therein, about wild plant populations, allows us to put forth the recommendations that in future landscape genomics studies, the experimental design should focus on increasing the total number of individuals sampled along the environmental heterogeneity under analysis^39^. In addition, we also suggest that researchers seek to evaluate the adaptation in wild plant populations using the Pool-Seq technique to increase the number of SNPs and accuracy in allele frequency estimates^52^. This technique has been used in population genomics as a powerful tool under different study scenarios, including model species^54^, allopolyploid species^55^ as well as for species with little or no genomic information available^56^. Following these guidelines, it will be possible to extrapolate findings from studies that are performed almost exclusively on species of temperate climate and expanding them to tropical species. In this way, landscape genomics can be conducted in tropical regions in relation to anthropogenic changes, in spite of existing budgetary constraints, and the data so generated can be used to develop strategies for management and conservation of biodiversity.
